# Human Ovarian Cancer Tissue Exhibits Increase of Mitochondrial Biogenesis and Cristae Remodeling

**DOI:** 10.3390/cancers11091350

**Published:** 2019-09-12

**Authors:** Anna Signorile, Domenico De Rasmo, Antonella Cormio, Clara Musicco, Roberta Rossi, Francesco Fortarezza, Luigi Leonardo Palese, Vera Loizzi, Leonardo Resta, Giovanni Scillitani, Ettore Cicinelli, Francesca Simonetti, Anna Ferretta, Silvia Russo, Antonio Tufaro, Gennaro Cormio

**Affiliations:** 1 Department of Basic Medical Sciences, Neurosciences and Sense Organs, University of Bari “Aldo Moro”, 70124 Bari, Italy; luigileonardo.palese@uniba.it (L.L.P.); silvia.russo92@gmail.com (S.R.); 2 CNR-Institute of Biomembranes, Bioenergetics and Molecular Biotechnologies, 70126 Bari, Italy; d.derasmo@ibiom.cnr.it (D.D.R.); c.musicco@ibiom.cnr.it (C.M.); a.ferretta@ibiom.cnr.it (A.F.); 3 Department of Biosciences, Biotechnologies and Biopharmaceutics, University of Bari “Aldo Moro” 70125 Bari, Italy; antonella.cormio@uniba.it; 4 Department of Emergency and Organ Transplantation, University of Bari “Aldo Moro”, 70124 Bari, Italy; roberta.rossi@uniba.it (R.R.); francescofortarezza.md@gmail.com (F.F.); leonardo.resta@uniba.it (L.R.); 5 Department of Biomedical Sciences and Medical Oncology, University of Bari “Aldo Moro”, 70124 Bari, Italy; vera.loizzi@uniba.it (V.L.); ettore.cicinelli@uniba.it (E.C.); francesca.simonetti@uniba.it (F.S.); gennaro.cormio@uniba.it (G.C.); 6 Department of Biology, University of Bari “Aldo Moro”, 70125 Bari, Italy; giovanni.scillitani@uniba.it; 7 Istituzional Biobank, Istituti di Ricovero e Cura a Carattere Scientifico (IRCCS) Istituto Tumori “Giovanni Paolo II” di Bari, Bari 70124, Italy; a.tufaro@oncologico.bari.it; 8 Gynecologic Oncology Unit, Istituti di Ricovero e Cura a Carattere Scientifico (IRCCS) Istituto Tumori “Giovanni Paolo II” di Bari, Bari 70124, Italy

**Keywords:** ovarian cancer, mitochondria, cAMP, mitochondrial biogenesis, oxidative phosphorylation system (OXPHOS), SIRT3, OPA1, prohibitins

## Abstract

Ovarian cancer (OC) is the most lethal gynecologic cancer characterized by an elevated apoptosis resistance that, potentially, leads to chemo-resistance in the recurrent disease. Mitochondrial oxidative phosphorylation was found altered in OC, and mitochondria were proposed as a target for therapy. Molecular evidence suggests that the deregulation of mitochondrial biogenesis, morphology, dynamics, and apoptosis is involved in carcinogenesis. However, these mitochondrial processes remain to be investigated in OC. Eighteen controls and 16 OC tissues (serous and mucinous) were collected. Enzymatic activities were performed spectrophotometrically, mitochondrial DNA (mtDNA) content was measured by real-time-PCR, protein levels were determined by Western blotting, and mitochondrial number and structure were measured by electron microscopy. Statistical analysis was performed using Student’s *t*-test, Mann-Whitney U test, and principal component analysis (PCA). We found, in OC, that increased mitochondrial number associated with increased peroxisome proliferator-activated receptor gamma coactivator 1-alpha (PGC1α) and mitochondrial transcription factor A (TFAM) protein levels, as well as mtDNA content. The OC mitochondria presented an increased maximum length, as well as reduced cristae width and junction diameter, associated with increased optic atrophy 1 protein (OPA1) and prohibitin 2 (PHB2) protein levels. In addition, in OC tissues, augmented cAMP and sirtuin 3 (SIRT3) protein levels were observed. PCA of the 25 analyzed biochemical parameters classified OC patients in a distinct group from controls. We highlight a “mitochondrial signature” in OC that could result from cooperation of the cAMP pathway with the SIRT3, OPA1, and PHB2 proteins.

## 1. Introduction

Ovarian cancer (OC) is the most lethal gynecologic cancer in Western countries and includes different histological subtypes, of which the large majority are diagnosed as serous and mucinous [1]. The absence of specific symptoms at the early stage of the disease leads to a delay of the diagnosis [1]; thus, disease recurrence is common. Indeed, OC cells are characterized by an elevated apoptosis resistance that, potentially, leads to chemo-resistance in the recurrent disease [2].

In general, metabolic changes are observed in cancer cells. Previously, the dependence on glycolysis in cancer cells was ascribed to defects in mitochondrial bioenergetics; however, recent data showed that mitochondria are functional in many cancer cells and they contribute, together with glycolysis, to sustain cell proliferation [3]. The mitochondrial oxidative phosphorylation system (OXPHOS) was found altered in OC [4,5], and mitochondria were proposed as a target for therapy [2]. Genetic and biochemical evidence suggests that, in addition to the OXPHOS alteration, the deregulations of mitochondrial biogenesis, morphology, dynamics (fusion/fission), and apoptosis represent key points involved in carcinogenesis [6,7]. However, the molecular actors at the bases of these mitochondrial processes remain to be further investigated in OC.

Mitochondrial biogenesis is the process via which cells increase their mitochondrial mass, and peroxisome proliferator-activated receptor gamma coactivator 1-alpha (PGC1α) is one of the master regulators of this process [8]. Recent articles showed altered expression of PGC1α in several tumors and metastasis [9]. The mitochondrial dynamics and structure depend essentially on optic atrophy 1 protein (OPA1) and dynamin-related protein 1 (DRP1) proteins, influencing several cellular processes in tumor cells such as apoptosis [10]. OPA1 also plays a role in resistance to apoptosis by regulating mitochondrial cristae remodeling [11]. Studies in OC cell cultures showed that the chemo-resistance to some drugs is partly due to a deregulation of OPA1 processing [12], leading to apoptosis resistance. In fact, OPA1 exists in long (L-OPA1) and short (S-OPA1) forms generated by proteolytic processing of L-OPA1 [13]. In response to a pro-apoptotic stimulus, the balance between the two forms changes in favor of S-OPA1, resulting in mitochondrial fragmentation and apoptosis [14]. OPA1 proteolytic processing is also modulated by its sirtuin 3 (SIRT3)-dependent deacetylation status [14,15] and prohibitin 2 (PHB2) [16]. Prohibitin proteins (PHB1 and PHB2) protect OPA1 from proteolytic processing in a chaperone-like manner [16,17]. SIRT3 is considered as a tumor promoter or suppressor depending on cell type. It was reported that the induction of apoptosis in the SKOV3 ovarian cancer cell line is associated with the activation of SIRT3 [18]; moreover, a decreased level of SIRT3 promotes the metastasis of OC [19]. SIRT3 is the main deacetylase within the mitochondria where it can be modulated by mitochondrial cAMP (mt-cAMP) [14]. Deregulation of the cAMP signal was found in KRAS tumor cells [20], and hypoxic activation of the cAMP/ protein kinase A (PKA) pathway was reported in different lines of cancer cells [21]. cAMP regulates mitochondrial respiratory chain activity and biogenesis [22,23,24,25,26], and it also plays an essential role in modulating apoptosis [27,28]. It was found that a decrease in mt-cAMP level could activate mitochondrial proteases, causing a decrease in SIRT3 protein level. This results in hyperacetylation of OPA1, which promotes its processing, pushing the cells toward apoptosis [14].

In this work, in human serious and mucinous OC tissues, we analyzed several aspects of mitochondrial biogenesis, dynamics, structure, and function in order to acquire molecular insights. We found an increased number of mitochondria, increased maximum mitochondria length, and reduced width and junction diameter of cristae, suggesting alteration of mitochondrial biogenesis, dynamics, and structure, respectively. The alteration of mitochondrial dynamics and structure was associated to an increased level of OPA1 and PHB2 proteins. Increased mitochondrial biogenesis was confirmed by increased PGC1α and mitochondrial transcription factor (TFAM) protein levels, as well as mitochondrial DNA (mtDNA) content. Analysis of respiratory chain complexes revealed a strong decrease in complex I activity. In addition, in OC tissues, we found activation of the cAMP/PKA pathway and an increased level of SIRT3 protein. Interestingly, principal component analysis (PCA) of the 25 analyzed biochemical parameters allowed classifying patients in a distinct group from control (CT) subjects, endorsing a “mitochondrial signature” in OC.

## 2. Results

### 2.1. OC Tissues Show an Increased Mitochondrial Number and Altered Mitochondrial Dynamics and Structure

**Figure 1:**
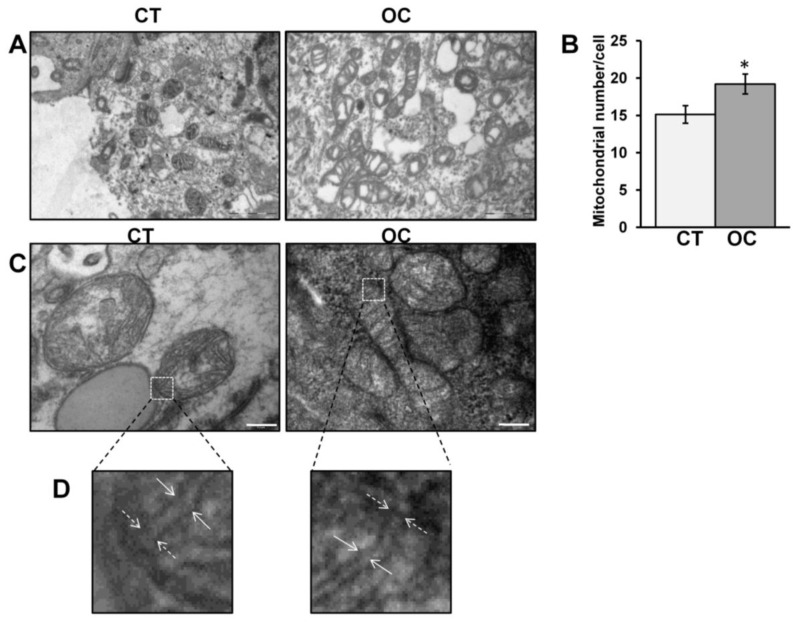
Electron microscopy images of mitochondria in control (CT) and ovarian cancer (OC) tissues. (**A**) Representative images of electron microscopy of semi-thin sections of CT and OC tissue samples (18,000×) (scale bar = 1 μm). (**B**) The histograms represent the means of values of mitochondria number ± standard error of the mean (SEM). (**C**) Representative images of electron microscopy of semi-thin sections of CT and OC tissue samples (56,000×) (scale bar = 0.2 μm) (* *p* < 0.05, Student’s *t*-test). For other details, see Section 4. (**D**) Magnification showing mitochondrial cristae width (solid arrows) and mitochondria cristae junction diameter (dashed arrows).

The study was conducted on 34 specimens: 18 controls (CT) and 16 OC tissues (histological subtypes serous and mucinous, grade G3). Fixed specimens of CT and OC were processed for electron microscopy (EM) analysis. The images obtained by EM showed an increased mitochondria number in OC with respect to CT tissues (Figure 1A,B) (also reported in Table 1, Table 2 and Table 3). To analyze the structural features of the mitochondria, the images were taken at higher magnification (Figure 1C).

The descriptive statistical analysis showed that OC mitochondria were characterized by an increased maximum length and a decreased cristae width and cristae junction diameter (Table 1, Table 2 and Table 3). The results of Shapiro-Wilk and Levene’s tests are in Table 2. With regard to the distribution, the Shapiro-Wilk test indicated that it did not deviate significantly from normality in both CT and OC tissue in terms of mitochondria number and cristae junction diameter. Normality was also supported in CT for cristae width. Levene’s tests indicated that the condition of homoscedasticity held only in mitochondria number and cristae width. Thus, we performed both a parametric (*t*-test) and a non-parametric (Mann-Whitney) test to exclude false positives due to the deviation of the distributions from normality. The results of *t*-tests with resampling and Mann-Whitney’s tests are in Table 3. Significant differences between CT and OC tissues resulted for each test.

**Figure 2:**
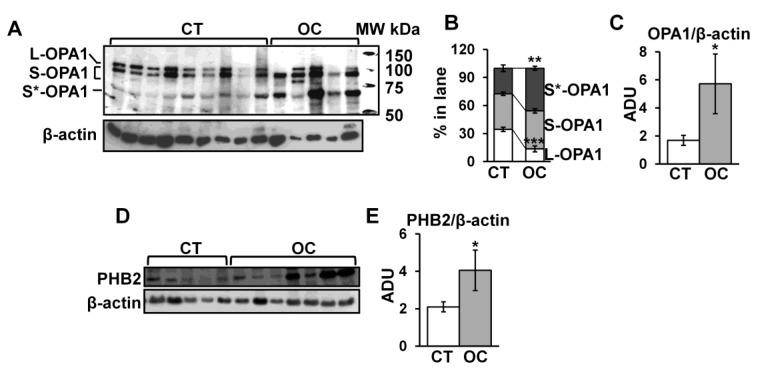
Optic atrophy 1 protein (OPA1) and prohibitin 2 (PHB2) protein levels in CT and OC tissues. The sample tissues of CT and OC were homogenized in buffer A (see Section 4) and centrifuged at 600× *g*; the resulting supernatant was sonicated and utilized for Western blotting analysis. (**A**,**D**) Representative images of Western blotting analysis. Proteins from CT and OC samples were loaded on 8% SDS-polyacrylamide gel electrophoresis (PAGE). After separation, the proteins were transferred on nitrocellulose membranes and immunoblotted with the antibodies against OPA1 and PHB2. Protein loading was assessed with β-actin antibody. The immunoblotting against β-actin antibody in panel D is the same shown in Figure 3D, belonging to the same experiment series. (**B**) The histograms represent the percentage of arbitrary densitometric units (ADU) of long (L), short (S), and S* forms of OPA1 in each lane. (**C**,**E**) The histograms represent the means of values of (arbitrary densitometric units) ADU ± SEM of samples. The values are means ± SEM of samples. (* *p* < 0.05, ** *p* < 0.01, *** *p* < 0.001, Student’s *t*-test). For other details, see Section 4. MW: molecular weight in kDa.

The increase in mitochondrial length and the decrease in crista width and crista junction diameter in OC tissues prompted us to investigate molecular aspects. The antibody against OPA1, in the CT group, immuno-revealed L and S forms at canonical molecular weight (Figure 2A). A further band at lower molecular weight (between 50 and 75 kDa), referred to as S* (Figure 2A), was detected in control subjects. In the OC group, the L form was decreased and almost undetectable in two subjects (Figure 2A,B). The amount of S form did not change significantly in the OC group with respect to CT (Figure 2A,B), while the S* form was found increased (Figure 2A,B). Densitometric analysis showed an increase in total level of OPA1 (L + S + S* forms) in OC with respect to CT tissues (Figure 2A,C). It was also reported that PHB2 exerts a role in OPA1 processing and apoptosis [16,17]. Western blotting and densitometric analysis of PHB2 revealed an increase in this protein in OC samples with respect to CT (Figure 2D,E, Supplementary material).

### 2.2. OC Tissues Show an Increase in Mitochondrial Biogenesis and a Decrease in Complex I Activity

**Figure 3:**
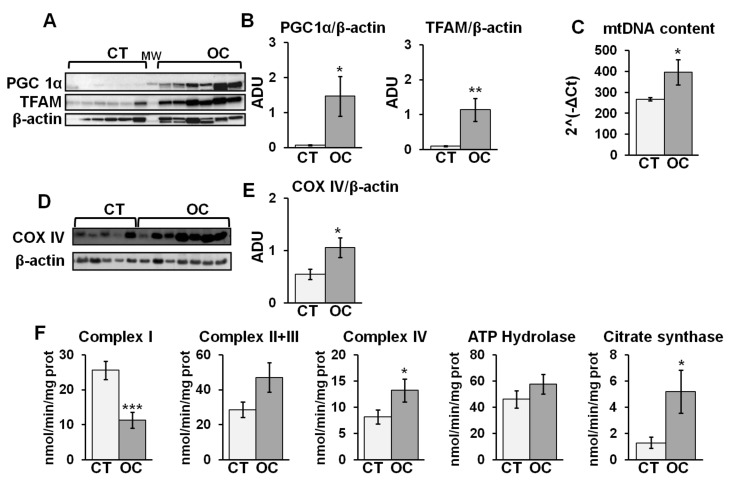
Mitochondrial biogenesis, oxidative phosphorylation system (OXPHOS), and citrate synthase activities in CT and OC tissues. The sample tissues of CT and OC were homogenized in buffer A and centrifuged at 600× *g*; the resulting supernatant was sonicated and utilized for Western blotting analysis and enzymatic activities. (**A**,**D**) Representative images of Western blotting analysis. Proteins from CT and OC samples were loaded on 8% SDS-PAGE. After separation, the proteins were transferred to nitrocellulose membranes and immunoblotted with the antibodies against peroxisome proliferator-activated receptor-gamma coactivator-1alpha (PGC1α), mitochondrial transcription factor A (TFAM), and subunit IV of cytochrome c oxidase (COX IV). Protein loading was assessed with β-actin antibody. (**B**,**E**) The histograms represent the means of values of ADU ± SEM of samples. (**C**) Mitochondrial DNA (mtDNA) content was calculated as target *ND1* gene and the reference β-actin gene. The histograms represent the average of the ratio ± SEM of values obtained from different samples. For mtDNA, 12 CT and 10 OC samples were analyzed. (**F**) The activities of OXPHOS complexes (complex I, II + III, IV, and ATP hydrolase) and citrate synthase were assayed spectrophotometrically. The histograms represent the means of values ± SEM of determinations in different samples (* *p* < 0.05, ** *p* < 0.01, *** *p* < 0.001, Student’s *t*-test). For other details, see Section 4.

The increase in mitochondrial number prompted us to investigate mitochondrial biogenesis and function. Thus, we analyzed the expression of proteins involved in mitochondrial biogenesis, PGC1α and TFAM, and the content of mtDNA. Figure 3A,B show a significant increase in PGC1α and TFAM protein levels in OC samples with respect to CT. An increased mtDNA/nuclear DNA (nDNA) ratio, evaluated by real time-PCR, was observed in OC compared to CT (Figure 3C). Western blotting and densitometric analysis of protein levels of subunit IV (COXIV) of cytochrome c oxidase showed a significant increase in COXIV protein level in OC samples with respect to CT (Figure 3D,E). In addition, we analyzed, in the homogenate fraction, by spectrophotometric assays, the activities of respiratory chain complexes and citrate synthase. A significant decrease in NADH-ubiquinone oxidoreductase (complex I) activity was observed in the OC tissues compared to CT (Figure 3F). On the contrary, the cytochrome c oxidase (complex IV) and citrate synthase activities were significantly higher in the OC samples compared to the CT (Figure 3F). No significant differences were found in succinate dehydrogenase + ubiquinol-cytochrome c reductase (complexes II + III) and ATP hydrolase activity (Figure 3F).

### 2.3. Increase in cAMP and SIRT3 Protein Level in OC Tissues

**Figure 4:**
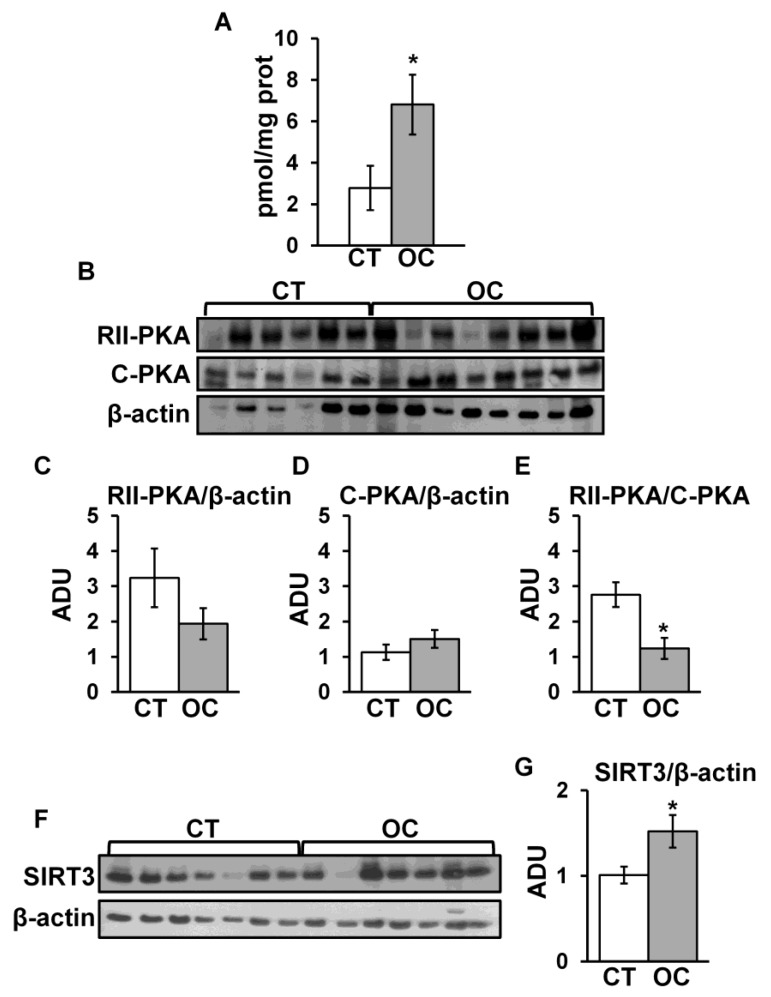
cAMP content, protein kinase A (PKA), and sirtuin 3 (SIRT3) protein levels in CT and OC tissues. (**A**) The specimens of control (CT) and ovarian cancer (OC) tissues were homogenized in 0.1 M HCl and centrifuged at 1000× *g*; the supernatant was used for determination of total cAMP level. The histograms represent the means of values ± SEM of samples. (**B**) The panel shows representative images of Western blotting analysis. The samples of CT and OC were homogenized in buffer A and sonicated; proteins were loaded on 8% SDS-PAGE. After separation, the proteins were transferred to nitrocellulose membranes and immunoblotted with the antibodies against the regulatory subunit of PKA (RII-PKA) and the catalytic subunit of PKA (C-PKA). Protein loading was assessed with β-actin antibody. (**C**,**D**) The histograms represent the means of values of ADU ± SEM of samples. (**E**) The histograms represent the means of values ± SEM of the RII-PKA/C-PKA ratio. For RII-PKA and C-PKA, 12 CT and 10 OC samples were analyzed. (**F**) Representative images of Western blotting analysis with antibody against SIRT3. Protein loading was assessed with β-actin antibody. (**G**) The histograms represent the means of values of ADU ± SEM of samples. For SIRT3, 14 CT and 16 OC samples were analyzed (* *p* < 0.05, Student’s *t*-test). For other details, see Section 4.

Furthermore, we investigated cAMP/PKA signaling and SIRT3, which can modulate mitochondrial biogenesis and dynamics [14,15,23]. Quantitative analysis in homogenate sample tissues of total cAMP level showed an increase in OC with respect to CT (Figure 4A). Therefore, we analyzed the expression level of the regulatory (RII) and catalytic (C) subunits of PKA. No significant difference was observed in the protein levels of RII-PKA and C-PKA in OC tissue samples with respect to CT (Figure 4B–D). However, a significant decrease in the RII-PKA/C-PKA ratio in OC with respect to CT samples was found, suggesting an increased activity of PKA in OC (Figure 4E) [29]. cAMP modulates the protein level and activity of SIRT3 [14,30]. Western blotting with a specific antibody against SIRT3 showed a significant increase in SIRT3 protein level in OC compared to CT tissues (Figure 4F,G).

### 2.4. PCA of 25 Analyzed Biochemical Parameters Allows Classifying OC Patients as a Distinct Group from Controls

**Figure 5:**
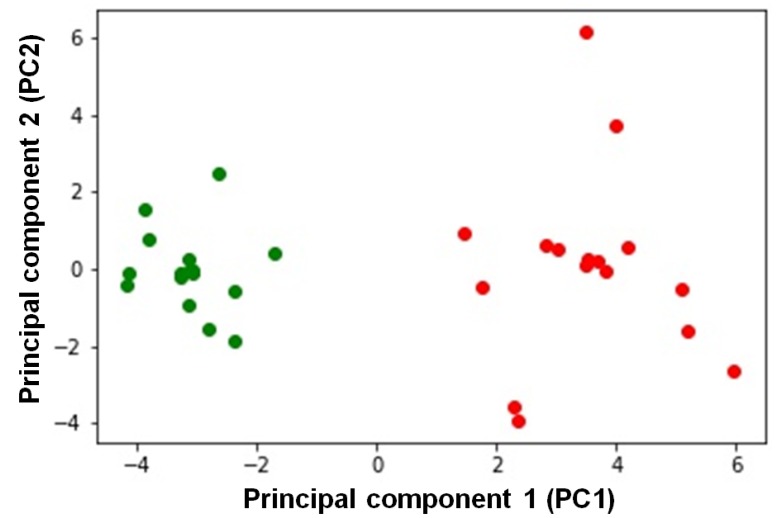
Principal component analysis. The figure reports the projection onto the first two principal components of the analyzed dataset. CT subjects are represented by green circles, while OC subjects are represented by red circles. For the analysis, the following parameters were used: complex I, cytochrome c oxidase, citrate synthase, complexes II + III, ATP hydrolase and citrate synthase activities, SIRT3/β-actin, PHB2/β-actin, COXIV/β-actin, cAMP level, L-OPA1/β-actin, S-OPA1/β-actin, S*-OPA1/β-actin, L- + S- + S*-OPA1/β-actin, % L-OPA1, % S-OPA1, % S*-OPA1, TFAM/β-actin, PGC1α/β-actin, mtDNA content, RII-PKA/β-actin, C-PKA/β-actin, RII-PKA/C-PKA, mitochondria number, and mitochondrial length. See Section 4 for details.

Finally, we performed an exploratory analysis on the whole dataset by PCA clustering (Figure 5). As can be easily appreciated by inspecting the figure, the control subjects and the affected ones formed two distinct groups. Note that the missing data were replaced by the intra-group averages. However, the clustering was quite robust, because the use of the overall averages for the missing data replacements led to few false negatives.

## 3. Discussion

More recent studies revealed a key role of mitochondria in tumorigenesis [31], for both cell proliferation and resistance to apoptosis. Moreover, cancer cells show high metabolic heterogeneity [5] and elevated apoptosis resistance; moreover, it was shown that they can switch to OXPHOS metabolism during the acquisition of therapy resistance [32]. This is particularly relevant in ovarian cancer [2], where only 10–30% of patients maintain a complete response to the initial therapy [33].

In this work, we analyzed mitochondria in human ovarian cancer (serous and mucinous) tissues in order to further understand the molecular mechanisms responsible for altered mitochondrial functions. First of all, we performed EM analysis to obtain information on mitochondrial morphology and number. EM analysis revealed, in OC with respect to CT, a decrease in cristae width and cristae junction diameter and an increase in mitochondrial length and mitochondrial number. This suggests an alteration of mitochondrial dynamics, structure, and biogenesis. To evaluate the molecular mechanism at the bases of these alterations, since deregulation of OPA1 processing was reported in OC [12], OPA1 protein level was analyzed. In fact, OC tissue showed an increased level of OPA1 protein in agreement with the increase in mitochondrial length observed in OC. Independently of its role in mitochondrial fusion, an increased level of OPA1 favors its own oligomerization at the cristae junction to reduce the cristae width at the junction, decreasing or delaying the release of apoptogenic molecules into the cytoplasm following cell death stimulus [34]. This is in agreement with the data presented here on morphometric analyses that revealed a decrease in cristae junction diameter in OC. This aspect could be related to resistance to apoptosis of these cancer cells. In addition, the electrophoretic profile of OPA1, in OC tissues, showed a decrease in L-form and an increase in S*-form (see Figure 2). The alternative S*-band could refer to the C-terminal fragment (CTF) of OPA1, already reported by others [35], derived by OPA1 C-terminus cleavage mediated by a still unknown cysteine protease [35]. The CTF fragment is associated with a decrease in complex I-dependent respiratory capacity and a drop in cristae density under nutrient starvation [35]. The authors speculated that this could represent an adaptive response of mitochondria to metabolic shift when the nutrients become limiting [35]. We show, for the first time, the presence of the CTF fragment of OPA1 in cancer tissue, and this might represent a metabolic adaptation of mitochondria to sustain cancer cell growth. OPA1 processing and stability is also controlled by PHB2 protein. In fact, loss of PHB2 is strongly associated with accelerated OPA1 processing, as well as impaired cell proliferation and apoptosis [16] while an over-expression of PHBs is reported to protect cells from apoptosis, and it was found in several tumor cells [16,36,37]. We found, in OC, an increased level of PHB2 associated with an increased level of OPA1, thus representing another element of resistance to apoptosis.

The enhanced mitochondria number in OC tissues can result in an increase in mitochondrial biogenesis. This finding is supported, at a molecular level, by the increase in PGC1α and TFAM protein levels, and the mitochondrial DNA content found in OC. PGC1α, a master regulator of mitochondrial biogenesis, promotes TFAM expression that, in turn, increases the transcription and translation of mitochondrial DNA [8,23]. Indeed, analysis of the mitochondrial respiratory chain showed an increase in complex IV activity in OC, associated with an increase in COXIV protein level and citrate synthase activity, supporting the increase in mitochondrial biogenesis. PHB2 can also induce mitochondrial biogenesis by promoting the expression of proteins encoded by both nuclear and mitochondrial DNA, via PGC-1α and TFAM, respectively [38,39]. Furthermore, PGC1α expression can also be regulated by activation of the cAMP/PKA pathway [40] and indirectly by SIRT3 [41,42]. We found, in OC tissues, a significant increase in cAMP level, associated with an activation of cAMP/PKA signaling. The activation of the cAMP/PKA pathway, found in OC, in addition to the activation of PGC1α [40], could participate in the stabilization of the SIRT3 protein [14], which in turn also activates PGC1α [41]. Noteworthy, in previous work, we showed that cAMP-mediated stabilization of SIRT3 prevented mitochondrial apoptosis by modulating OPA1 acetylation/processing [14]. Thus, the presented data suggest a role of cAMP and SIRT3 in modulating mitochondrial biogenesis, morphology, and resistance toward apoptosis in OC cells. Furthermore, cAMP and SIRT3 also regulate complex I activity [24,25,43]. Despite the increase in mitochondrial biogenesis, we found a significant decrease in complex I activity. Preliminary data indicated that the reduction in activity is not due to a defect in complex I assembly/structure; in fact, blue native gel electrophoresis, followed by SDS gel electrophoresis, indicated an increased number of OXPHOS complexes in OC with respect to CT [44]. In the field of the cancer metabolic restructuring [3], loss of complex I function was reported in different human tumors, also associated with increased cAMP level [20], and often related to mtDNA mutations, and this is generally accepted as a tumor growth contributor [3,6,45]. In addition, Miwa and collaborators reported, for PHB, a role in the stabilization of partially assembled sub-complex I responsible for the defective function of fully assembled complex I [46]. The increased level of PHB2 in OC, in addition to its role in resistance to apoptosis, could also be responsible for the stabilization of a sub-complex I, possibly contributing to the decrease in complex I activity in ovarian cancer tissue.

Summarizing, we show that serous and mucinous histological types of ovarian cancer tissues are characterized by increases in mitochondrial biogenesis and cristae remodeling. This could be the result of activation of cAMP/PKA and stabilization of SIRT3. In addition, the increased level of SIRT3 works together the increased level of PHB2 for the stabilization of OPA1, which, in addition to its role in dynamics, also controls mitochondrial morphology, keeping the cristae junction narrow, giving more resistance toward apoptosis [16,34]. However, both cAMP and SIRT3, which normally promote the activity of complex I [24,43], failed to promote it in OC.

Interestingly, PCA of the 25 analyzed biochemical parameters allowed classifying OC patients as a distinct group from controls. We highlight, in the heterogeneity of ovarian cancer, a characteristic “mitochondrial signature”, characterized by increased mitochondrial biogenesis and altered mitochondrial structure that could result from the cooperation of cAMP pathway and the SIRT3, OPA1, and PHB2 proteins. The molecular parameters that define the OC group are interconnected among them and support a “mitochondrial signature”.

## 4. Materials and Methods 

### 4.1. Patient Samples

The study was conducted on 34 subjects: 16 patients with ovarian cancer (histological subtypes serous and mucinous, grade G3) and 18 control cases (CT). The control cases were represented by six samples of ovarian tissue and 12 samples of tubal fimbria tissues that are considered the histological origin of ovarian cancer cells [47]. For all data, no significant difference was observed between ovary and tubal fimbria tissues in the CT group and between mucinous and serous carcinoma in the OC group. The OC tissue samples were taken during surgery to remove the tumor mass, while the CT samples were taken during surgery for non-tumor diseases. Patients and control cases were selected by the Department of Gynecology at the University of Bari Policlinico. Tissue samples were taken and frozen at −80 °C. None of the patients received any treatment (radiotherapy, chemotherapy, or hormone therapy) before surgery. The study was approved by the Independent Ethical Committee—IEC—Azienda Ospedale Consorziale Policlinico, Bari (n. 3574), and all patient and control subjects signed informed consent. All used methods were performed in accordance with the ethical standards of the institutional and national research committee and with the 1964 Helsinki declaration and its later amendments or comparable ethical standards.

### 4.2. Electron Microscopy

Samples were fixed in 2.5% glutaraldehyde and, after an overnight wash in the same buffer, the samples were post-fixed with 1% osmium tetroxide in PBS for 2 h at 4 °C. Fixed specimens were processed for embedding in Epoxy Resin-Araldite(M) CY212 (TAAB, Aldermaston, UK). Semi-thin 2-µm-thick sections were stained with Toluidine blue. Ultra-thin sections were mounted on Formvar-coated nickel grids and stained routinely with uranyl acetate and lead citrate. Images of semi-thin sections were captured using a Nikon photomicroscope equipped with a Nikon Digital sight DS-U1 camera (Nikon Instruments SpA, Calenzano, Italy). Ultra-thin sections were observed using a transmission electron microscope Morgagni 268 (FEI Company, Milan, Italy). The mitochondria were chosen in the mitochondria aggregates in the different areas of cells. The count was performed at 18,000× by using the “touch count” function of the ITEM-SIS (SOFT IMAGING SYSTEM-Olympus) program of the electron transmission microscope. The mitochondria length and the mitochondria cristae parameters (width and junction diameter) were measured at 56,000× and 140,000×, respectively, by using the “arbitrary line” function of the electronic transmission microscope. The data regarding mitochondria number were obtained examining three fields for each sample. For length of mitochondria, 12 mitochondria for each sample were examined. For cristae width, five mitochondria for each sample were examined. For cristae junction diameter, three mitochondria for each sample were examined. All measurements were designed with the software function, indicating the two desired points and the software pressure.

### 4.3. Electrophoretic Procedures and Western Blotting

The sample tissues (100–400 mg) were homogenized in 0.25 M mannitol, 10 mM Tris, and 0.25 mM phenylmethylsulfonyl fluoride (PMSF) (Buffer A) and sonicated at 30% amperage for 20 s. The proteins were separated by 8% SDS-PAGE and transferred to a nitrocellulose membrane. The membrane was blocked with 5% fatty-acid-free dry milk in 500 mM NaCl, 20 mM Tris, 0.05% Tween-20 (pH 7.4; TTBS) for 3 h at 4 °C and probed with antibodies against OPA1 and COXIV (Thermo scientific, Pierce Antibodies, Lausanne Switzerland), PHB2 (Invitrogen, Paisley, UK), RIIα-PKA, C-PKA, TFAM (Santa Cruz Biotechnology, Heidelberg, Germany), SIRT3 (Cell Signalling, Danvers, MA, USA), PGC1α (Abcam Cambridge, UK), and β-actin (Sigma-Aldrich, St. Louis, MO, USA). After being washed in TTBS, the membrane was incubated for 60 min with anti-rabbit or anti-mouse IgG peroxidase-conjugated antibodies. Immunodetection was then performed, after further TTBS washes, with enhanced chemiluminescence (ECL) (Euroclone, Paignton, UK). Image acquisition was performed by the ChemiDoc imaging system (BioRad, Milan, Italy), and densitometric analysis was performed by the Image Lab software (BioRad, Milan, Italy).

### 4.4. Quantification of mtDNA

Total DNA was prepared from 20 mg of tissues using a Wizard Genomic DNA Purification Kit (Promega). MtDNA content was measured by real-time PCR using a QuantStudio 7 Flex real-time PCR (Applied Biosystems). The primers for the mtDNA were ND1-forward (For) (5’–CCCTAAAACCCGCCACATCT–3’) and ND1-reverse (Rev) (5’–TAGAAGAGCGATGGTGAGAGCTAA–3’) and were used together with the mtDNA probe (5’–FAM–CCATCACCCTCTACATCACCGGCC–TAMRA–3’). The primers for β-actin were Actin-For (5’–CCCAGCCATGTACGTTGCTA–3’) and Actin-Rev (5’–CGTCACCGGAGTCCATCAC–3’), and the probe was (5’–FAM–ACGCCTCTGGCCGTACCACTGG-TAMRA–3’. The method was validated by evaluating the equal reaction efficiency of the two amplicons. Each sample was analyzed in triplicate. The PCR was performed separately for the target *ND1* gene and the reference β-actin gene. The PCR mixture contained the specific primers (800 nM), specific TaqMan probes (200 nM), 5–10 ng of DNA, and 1× Taqman Fast Advanced MMIX (Life Technologies). Amplification conditions were 50 °C for 2 min, 95 °C for 2 min, and 40 cycles of 95 °C for 1s and 60 °C for 20 s. The difference in threshold cycle values ΔCt, namely, CtND1/CtActin, was used as a measure of the relative abundance of the mitochondrial genome. In particular, the mtDNA/nDNA ratio is reported as 2^−ΔCt^.

### 4.5. Enzymatic Spectrophotometric Assays

For enzymatic spectrophotometric assays, tissue samples (100–400 mg) were homogenized in Buffer A. The homogenate was then centrifuged at 600× *g* for 10 min at 4°C; the resulting supernatant was sonicated at 30% amperage for 20 s. The obtained fraction was frozen at −80 °C before carrying out the subsequent analyzes.

The NADH-UQ oxidoreductase activity (complex I) was performed in 40 mM potassium phosphate buffer, pH 7.4, 5 mM MgCl_2_, in the presence of 3 mM KCN, 1 μg/mL antimycin, and 200 μM decylubiquinone, using 70 μg of protein, by following the oxidation of 100 μM NADH at 340–425 nm (Δε = 6.81 mM^−1^∙cm^−1^). The activity was corrected for the residual activity measured in the presence of 1μg/mL rotenone.

Succinate-cytochrome c oxidoreductase (complex II + III) activity was performed in 25 mM potassium phosphate buffer, pH 7.4, 5 mM MgCl_2_ in the presence of 20 mM succinate, 2 mM KCN, 65 μM decylubiquinone, and 20 μM cytochrome c, using 50 μg of protein. The cytochrome c reduction was followed at 550–540 nm (Δε = 19.1 mM^−1^∙cm^−1^).

Cytochrome c oxidase (complex IV) activity was measured by following the oxidation of 10 μM cytochrome c at 550–540 nm (Δε = 19.1 mM^−1^∙cm^−1^). Enzymatic activity was measured in 10 mM phosphate buffer, pH 7.4, using 50 μg of protein. This rate was inhibited over 95% by 2 mM KCN.

ATP hydrolase activity was measured by an ATP-regenerating system. Firstly, 100 μg of protein was suspended in a buffer consisting of 375 mM sucrose, 75 mM KCl, 30 mM Tris-HCl pH 7.4, 3 mM MgCl_2_, 2 mM PEP, 55 U/mL lactate dehydrogenase, 40 U/mL pyruvate kinase, and 0.3 mM NADH. The reaction was started by the addition of 1 mM ATP, and the oxidation of NADH was followed at 340–425 nm (Δε = 6.81 mM^−1^∙cm^−1^).

The citrate synthase activity was measured using 50 μg of protein in a solution containing 0.1 M Tris pH 8, 0.2% Triton X-100 in the presence of 0.5 mM 5,5′-Dithiobis(2-nitrobenzoic acid) (DTNB), 0.5 mM Acetyl-CoA, and 0.5 mM oxaloacetate; the reaction was followed at 419 nm.

### 4.6. cAMP Assay

For analysis of cAMP level, the tissue samples (80 mg) were homogenized in 0.1 M HCl (1:10 *w*/*v*), centrifuged at 1000× *g* for 10 min. The supernatants were used to determine cAMP concentration using a direct immunoassay kit (Assay Designs) as described by the manufacturer. Total protein concentration was determined by BioRad protein assay. The cAMP levels in the samples were normalized to the total protein concentration and expressed as pmol/mg protein.

### 4.7. Data Analysis

All data presented in Figure 1, Figure 2 and Figure 3 are means ± SEM (standard error of the mean). Statistical difference was determined by Student’s *t*-test. A *p*-value < 0.05 was considered as statistically significant (* *p* < 0.05; ** *p* < 0.01; *** *p* < 0.001).

The most common descriptive statistics (mean, standard error, variance, kurtosis, skewness, and range) were computed for the mitochondria number, mitochondrial maximum length, and cristae junction diameter in both the CT and OC samples. Shapiro–Wilk tests were performed for each dataset to assess significant deviations from normal distribution. Levene’s tests for homogeneity of variances were also performed between CT and OC for each variable. Significant differences were estimated by Student’s *t*-tests, followed by resampling through a bootstrap of *t*-values (10,000 iterations) and by non-parametric Mann-Whitney’s tests. Statistical computations were generated by the Real Statistics Resource Pack software (Release 4.3) [48]. For principal component analysis (PCA) and random projection, data preprocessing and numerical manipulations were performed essentially as described in References [49,50]. Missing experimental data were replaced by the intra-group averages.

## 5. Conclusions

In this work, we performed an analysis of mitochondria in human tissues of serous and mucinous ovarian cancers. We showed that OC tissues exhibit an increased mitochondrial biogenesis and altered cristae structure. The observed increase in mitochondrial biogenesis, in OC, is in agreement with recent works showing an increase in mitochondrial biogenesis in cancer cells [5,32]. However, while several cancer cells, with higher dependence on OXPHOS, appear to be resistant to conventional therapy [32] associated with increased sensitivity to complex I inhibitors [32], OC cells, with higher metabolic dependence on OXPHOS, were reported to be sensitive [5] and also insensitive [2] to conventional therapy in agreement with the metabolic heterogeneity of OC [2,5]. In this context, it should be recalled that only a small percentage of OC responds to the initial therapy [33]. In light of this, the “mitochondrial signature”, characterized by a decrease in complex I activity, despite the increased biogenesis in OC, could represent different possible cues to drug targets.

## Figures and Tables

**Table 1:** Descriptive statistics for the mitochondria number, maximum length, cristae width, and cristae junction diameter in controls (CT) and ovarian cancer (OC).

Statistics	Mitochondria Number		Mitochondria Maximum Length (nm)		Cristae Width (nm)		Cristae Junction Diameter (nm)	
	CT	OC	CT	OC	CT	OC	CT	OC
**Mean**	15.13	19.17	543.97	707.06	24.07	21.24	25.15	22.46
**Standard Error**	1.35	1.11	13.90	27.82	0.28	0.22	0.91	0.31
**Sample Variance**	54.33	35.50	23,184.61	83,577.99	11.63	12.21	16.56	7.45
**Kurtosis**	−0.56	1.20	−0.41	2.07	−0.19	0.35	−0.99	−0.04
**Skewness**	0.21	0.43	0.38	1.28	0.14	0.92	−0.16	0.32
**Range**	29	28	638.73	1429.22	15.95	17.44	14.29	13.69

**Table 2:** Probabilities associated with Shapiro-Wilk and Levene’s tests for mitochondria number, maximum length, cristae width, and cristae junction diameter in controls (CT) and ovarian cancer (OC).

Statistics	Mitochondria Number		Mitochondria Maximum Length		Cristae Width		Cristae Junction Diameter	
	CT	OC	CT	OC	CT	OC	CT	OC
Shapiro-Wilk	0.46	0.34	0.014 *	0.000 **	0.46	0.000 **	0.67	0.35
Levene		0.070		0.000 **		0.815		0.006 **

* *p* < 0.05; ** *p* < 0.01.

**Table 3:** Student’s *t*-test and Mann-Whitney’s test for mitochondria number, maximum length, cristae width, and cristae junction diameter in controls (CT) and ovarian cancer (OC).

Statistics	Mitochondria Number			Mitochondria Maximum Length			Cristae Width			Cristae Junction Diameter		
	Value	df	*p*	Value	df	*p*	Value	df	*p*	Value	df	*p*
Student’s *t*-test (st.err.)	2.310(1.75)	57	0.012 *	5.404(30.18)	226	0.000 **	7.912(0.36)	398	0.000 **	3.554 (0.76)	98	0.000 **
Mann-Whitney U test	303	-	0.022 *	4111.5	-	0.000 **	9914.5	-	0.000 **	480.0	-	0.003 **

st.err. = standard error; df = degrees of freedom. * *p* < 0.05; ** *p* < 0.01.

## Supplementary Materials

The following are available online at https://www.mdpi.com/2072-6694/11/9/1350/s1.
