# Supplementary material for: Human Ovarian Cancer Tissue Exhibits Increase of Mitochondrial Biogenesis and Cristae Remodeling
Source: Cancers (Basel). 2019 Sep 12;11(9):1350. doi: 10.3390/cancers11091350 (PMC6770021; doi:10.3390/cancers11091350)
Supplement: Supplementary file 1 [file cancers-11-01350-s001.pdf]

Supplementary figure 1 (S1)

A

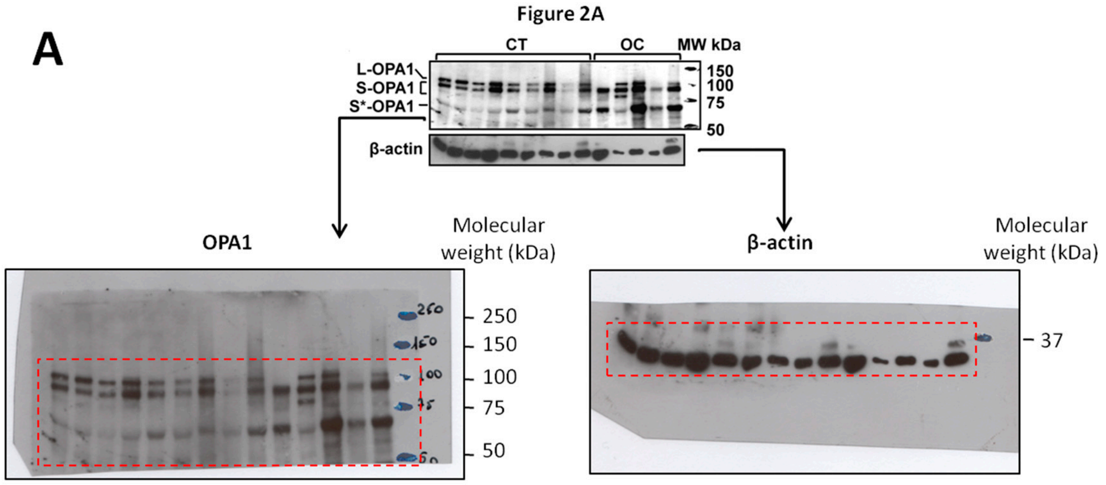

OPA1	BAND	CT									OC				
	L1	0,760	0,748	0,571	0,829	0,593	0,545	1,144	0,195	0,536	0,123	2,434	2,035	0,431	0,689
S	S1	0,793	0,526	0,553	0,938	0,735	0,659	1,429	0,605	0,688	1,174	4,261	2,581	2,133	1,412
	S2	nd	nd	nd	nd	nd	nd	nd	nd	nd	nd	2,237	nd	nd	nd
	S*	0,336	0,211	0,147	0,203	0,667	0,501	0,989	1,584	0,847	1,699	3,125	5,525	4,799	2,593

nd: not detectable

B

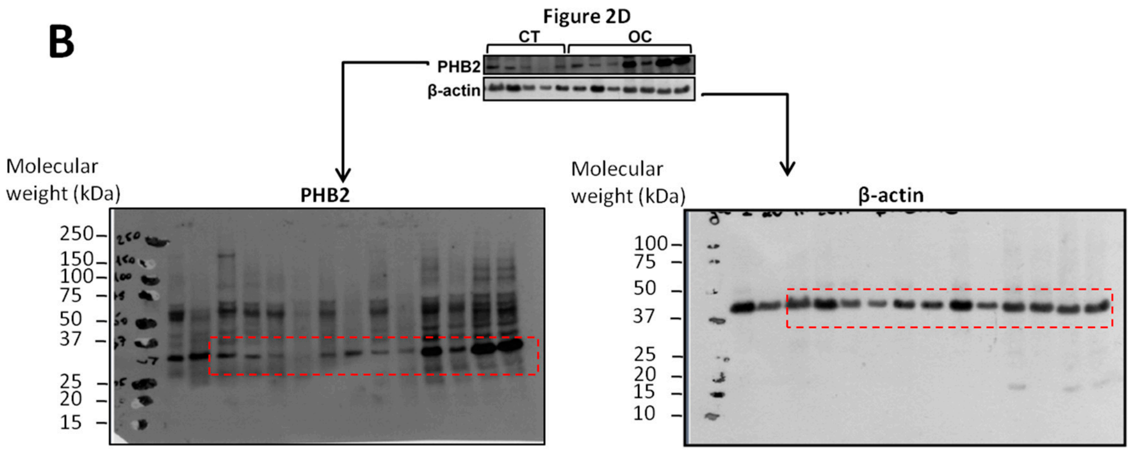

	CT					OC						
PHB2	2,223	3,311	3,284	1,088	1,414	1,074	2,078	3,229	6,509	3,817	12,603	9,164

Figure S1. Raw data for western blotting in control (CT) and ovarian cancer (OC) groups. (A) Full scan of western blots of OPA1 and β-actin shown in Figure 2A. Blot was cut between 50 and 37 kDa markers. Cropped areas are marked by red color. The table shows the densitometric analysis of OPA1 western blot (normalized to loading control). (B) Full scan of western blots of PHB2 and β-actin shown in Figure 2D. Cropped areas are marked by red color. The table shows the densitometric analysis of PHB2 western blot (normalized to loading control).

Supplementary figure 2 (S2)

A

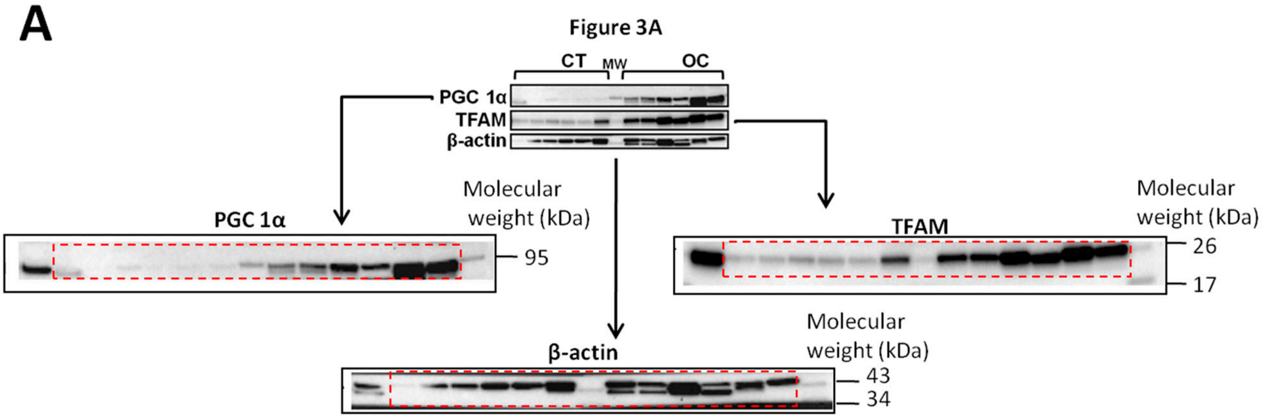

	CT						OC					
PGC1α	0,04	0,06	0,13	0,09	0,1	0,12	0,29	0,37	1,01	1,54	5,15	0,61
TFAM	0,06	0,04	0,16	0,09	0,18	0,18	0,67	0,42	0,62	1,64	2,01	0,51

B

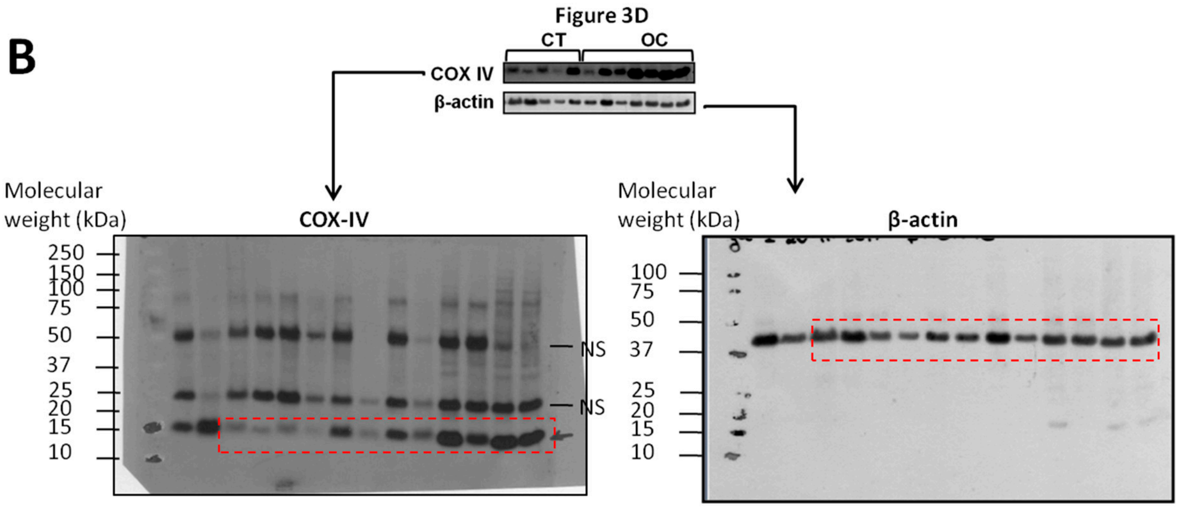

	CT					OC						
COXIV	0,574	1,156	0,636	0,447	1,023	0,368	0,677	1,088	1,216	1,191	1,440	1,572

Figure S2. Raw data for western blotting in control (CT) and ovarian cancer (OC) groups. (A) Full scan of Western blots of PGC1α, TFAM and β-actin shown in Figure 3A. Membrane was cut between 95 and 72 kDa markers (PGC1α, left panel), between 43 and 55 and between 34 and 26 kDa markers (β-actin, middle panel) and 26 and 34 kDa markers (TFAM, right panel). Cropped areas are marked by red color. The table shows the densitometric analysis of PGC1α and TFAM western blots (normalized to loading control). NS: nonspecific. (B) Full scan of western blots of COX-IV and β-actin shown in Figure 3D. Cropped areas are marked by red color. The table shows the densitometric analysis of COX-IV western blot (normalized to loading control).

Supplementary figure 3 (S3)

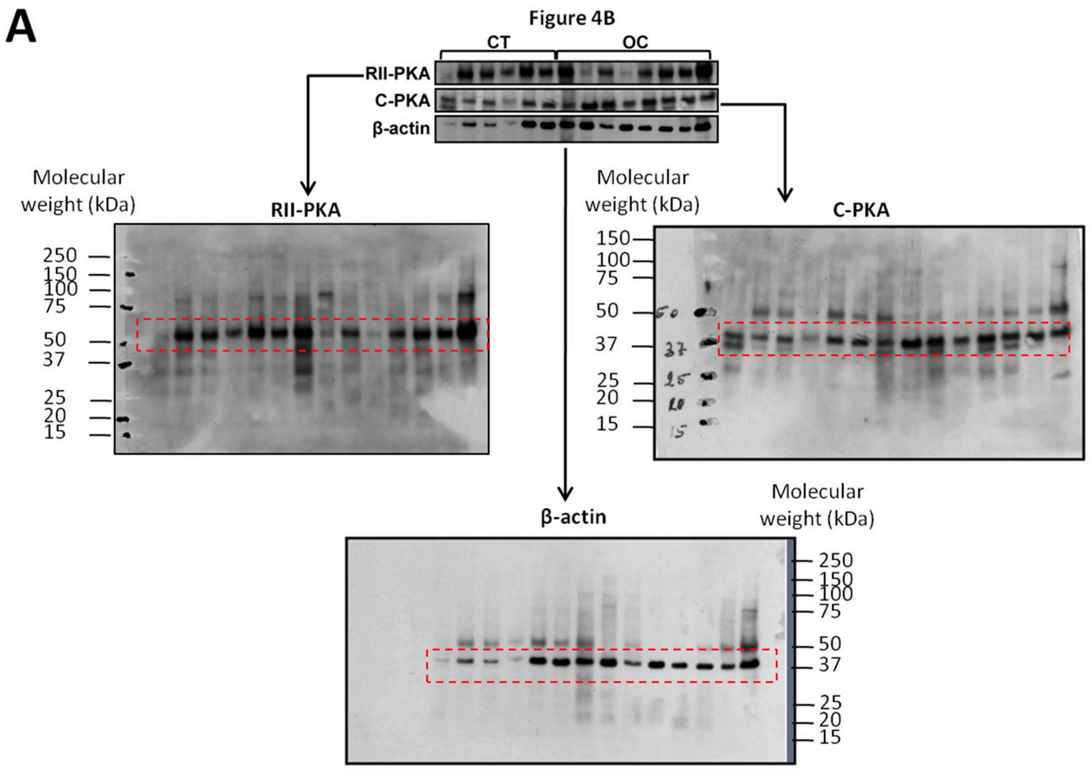

	CT						OC							
RII-PKA	2,024	3,158	4,040	5,931	1,764	1,288	1,780	0,462	2,041	0,196	2,003	2,359	2,362	1,811
C-PKA	4,689	0,855	1,582	1,734	0,753	0,719	0,832	1,163	2,278	0,770	1,815	1,316	1,354	0,772

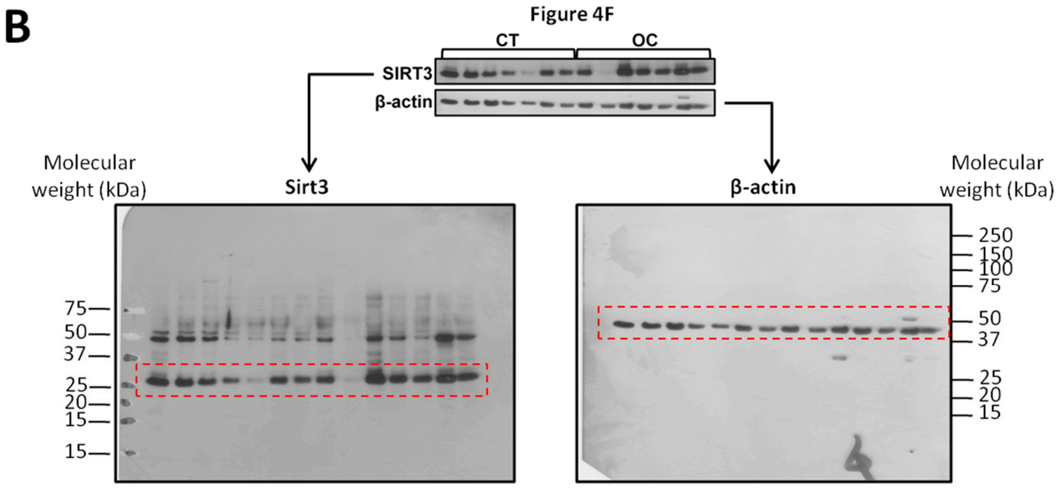

	CT							OC						
SIRT3	1,160	1,148	0,836	1,108	0,686	1,236	1,003	1,633	0,888	1,662	2,065	1,693	1,048	1,128

Figure S3. Raw data for western blotting in control (CT) and ovarian cancer (OC) groups. (A) Full scan of Western blots of RII-PKA, C-PKA and β-actin shown in Figure 4B. Cropped areas are marked by red color. The table shows the densitometric analysis of RII-PKA and C-PKA western blots (normalized to loading control). (B) Full scan of Western blots of SIRT3 and β-actin shown in Figure 4F. Cropped areas are marked by red color. The table shows the densitometric analysis of SIRT3 western blot (normalized to loading control).
